# Referral management centres as a means of reducing outpatients attendances: how do they work and what influences successful implementation and perceived effectiveness?

**DOI:** 10.1186/s12875-016-0434-y

**Published:** 2016-03-24

**Authors:** Sarah L. Ball, Joanne Greenhalgh, Martin Roland

**Affiliations:** RAND Europe, Westbrook Centre, Milton Road, Cambridge, CB4 1YG UK; School of Sociology and Social Policy, University of Leeds, LEEDS, LS2 9JT UK; Cambridge Centre for Health Services Research, Institute of Public Health, University of Cambridge School of Clinical Medicine, Forvie Site, Robinson Way, Cambridge, CB2 0SR UK

## Abstract

**Background:**

The rising volume of referrals to secondary care is a continuing concern in the NHS in England, with considerable resource implications. Referral management centres (RMCs) are one of a range of initiatives brought in to curtail this rise, but there is currently limited evidence for their effectiveness, and little is known about their mechanisms of action. This study aimed to gain a better understanding of how RMCs operate and the factors contributing to the achievement of their goals. Drawing on the principles of realist evaluation, we sought to elicit programme theories (the ideas and assumptions about how a programme works) and to identify the key issues to be considered when establishing or evaluating such schemes.

**Methods:**

Qualitative study with a purposive sample of health professionals and managers involved in the commissioning, set-up and running of four referral management centres in England and with GPs referring through these centres. Semi-structured interviews were conducted with 18 participants. Interviews were audio-recorded and transcribed. Data were analysed thematically.

**Results:**

Interview data highlighted the diverse aims and functions of RMCs, reflecting a range of underlying programme theories. These included the overarching theory that RMCs work by ensuring the best use of limited resources and three sub-theories, relating to how this could be achieved, namely, improving the quality of referrals and patient care, reducing referrals, and increasing efficiency in the referral process. The aims of the schemes, however, varied between sites and between stakeholders, and evolved significantly over time. Three themes were identified relating to the context in which RMCs were implemented and managed: the impact of practical and administrative difficulties; the importance and challenge of stakeholder buy-in; and the dependence of perceived effectiveness on the aims and priorities of the scheme. Many RMCs were described as successful by those involved, despite limited evidence of reduced referrals or cost-savings.

**Conclusions:**

The findings of this study have a number of implications for the development of similar schemes, with respect to the need to ensure clarity of aims and to identify indicators of success from the outset, to anticipate scheme evolution and plan for potential changes with respect to IT systems and referral processes. Also identified, is the need for further research that evaluates the effectiveness and cost effectiveness of particular models of RMC.

## Background

The rising volume of referrals to secondary care and associated resource implications is a continuing concern for the NHS in England. There were 75.5 million outpatients attendances in 2012/13, a rise of 25 % in six years [1]. The introduction of referral management centres (RMCs) is one of a range of measures taken to tackle this issue, by seeking to manage the referral process. The interface between primary and secondary care is a central organisational feature of the NHS and many other health-care systems. In the UK, general practitioners (GPs) fulfil a ‘gatekeeper’ role, determining which patients require access to specialist care. While similar models operate in Australia, Denmark and the Netherlands, other countries, such as France, Germany and some parts of the United States’ health care system use less formalised referral systems, which provide financial or other incentives to encourage (but not mandate) patients to seek an opinion from a primary care practitioner before seeing a specialist. Worldwide, a range of factors including shifting demographics, changing patient expectations and the increasing burden of chronic disease, are leading to increased demand and healthcare costs, resulting in the adoption of a range of referral management strategies, targeting primary care, specialist services or infrastructure (with RMCs falling into the latter category) [2]. A recent review found that at present, most published literature on RMCs relates to interventions implemented within the UK [3].

The use of RMCs in England has not been prescribed in formal policy papers, but is an approach that has been developed by primary care trusts (and later clinical commissioning groups; CCGs) to help them address the challenges they face. RMCs lie at the active end of a continuum of referral management approaches, which also includes peer review of referrals, and the use of referral guidelines [4] or priority scores (such as those that have been implemented in New Zealand and Canada to prioritise patients for elective procedures) [5, 6]. Broadly, the role of the RMC is to act as an external arbiter to review referrals and to perform some action with respect to them (i.e. to reject, divert, or provide advice or some additional function) but considerable diversity in their form and organisation is acknowledged in the literature [7]. While some RMCs are designed primarily to manage bookings or facilitate patient choice, many have among their aims the clear implicit or explicit goal of reducing referrals. This study focusses on the latter subset of initiatives.

Widespread concerns have been expressed in the academic, GP and UK national press [7–12] regarding the potential negative effects of RMCs, such as the introduction of error and delay into the referral process, and objections have been made both to the undue interference of managers in clinical decisions and to the involvement of clinicians other than doctors in the review process. In addition, the limited evidence published on their effectiveness is equivocal and suggests that reduction in referrals by RMCs is less likely to represent value for money than the use of more passive alternatives such as peer review [4, 13, 14]. Despite this, RMCs still represent a widely used approach and new schemes continue to be developed across the UK. Around a quarter of CCGs were reported to be using a RMC in 2014, 64 % of which had been set up since 2010 and 21 % since CCGs took control of commissioning in 2013 [15]. Their continued popularity in the absence of supportive evidence raises questions regarding the rationale behind their implementation.

The epitome of a complex intervention, RMCs rely on human agency and the actions of multiple stakeholders to make them work, require a sequence of intervening processes to occur before they achieve their ultimate outcome and furthermore, they have a tendency to adapt and evolve over time [16]. Indeed, a recent review of demand management interventions demonstrated that such programmes require concomitant changes at all levels of the health system to make them work effectively, and that they inevitably evolve over time as stakeholders make changes in response to experiences of what does and does not work on the ground [17].

This study sought to gain a better understanding of the inner workings of RMCs and the factors contributing to the achievement of their goals. To begin to understand this complexity, we have drawn on the principles of realist evaluation [18], an approach premised on the idea that complex interventions represent *theories* (held by those designing and implementing them). While a full-scale realist evaluation of RMCs, (involving developing, testing and refining theories, to explain why they work in some circumstances and not others), was beyond the scope of this study, we sought to engage in the first stage of this process, that of eliciting programme theories (the ideas and assumptions about what a programme is intended to achieve and how it is supposed to work). In so doing, we aimed to identify the key issues to be considered when establishing or evaluating such schemes.

## Methods

The study took place at four RMC sites in dispersed geographic areas in England, UK. We identified potentially eligible RMCs through internet search and via the authors’ professional networks. A broad internet search for ‘referral management’ (using Google) yielded a limited number of references to specific referral management ‘centres’, ‘services’ or ‘schemes’ in England. Emails were sent to contacts at all RMC sites for which contact details were available online (*n* = 8) and to three sites identified through professional networks. Advertisement in the GP press yielded no further responses. All sites expressing an interest were found to be eligible to take part (meeting the inclusion criterion of having been ‘set up with the implicit or explicit aim of reducing referrals’) and were thus included.

While we had initially aimed to include two schemes deemed to be successful and two that had experienced problems, limited response to invitations to participate meant that schemes were not selected on this basis specifically. It became apparent through the course of the interviews, however, that success could be defined on many levels and that the evolving nature of such schemes had resulted in identified problems and implemented solutions for three of the included schemes, the fourth although deemed successful with respect to reduced referrals, had closed down. Thus the selected schemes covered a range of degrees of success and sustainability.

### Sampling and data collection

Purposive sampling was used to select participants involved in the commissioning, set-up and running of the identified RMCs and GP referrers. The study sought to include clinicians and managers with a variety of roles to gain a wide range of perspectives. Individuals fulfilling these roles were approached via email describing the purpose of the study and inviting them to participate. All those approached agreed to participate. In-depth interviews were conducted by two researchers (SB and MR) at the participant’s workplace or by telephone, between July 2013 and May 2014. Verbal consent was sought prior to interview. A common interview guide was used for each interview, although emphasis was given to allowing participants to talk from their own perspective. Questions were derived from issues identified in the literature regarding how the RMCs were intended to work and the contextual factors that influenced this. Questions were designed to understand the intended aims of the schemes, how stakeholders felt about the schemes and what had worked well and less well with respect to their implementation. Topics covered included: the design of the RMC; its aims; changes in the scheme since it was initiated; acceptability to a range of stakeholders; outcomes; and local context. Interviews took the form of a guided conversation, during which programme theories relating to RMCs (identified through relevant literature and through preceding interviews) were explored. The interview schedule developed iteratively. Interviews were audio-recorded and transcribed verbatim. Transcripts were anonymised through removal of references to identifiable names and places.

### Data analysis

Data analysis proceeded simultaneously with data collection and informed the iterative development of the interview schedule. Thematic analysis of the data was conducted based on the principles outlined by Boyatzis [19]. Transcripts were read and re-read and ‘codes’ applied to meaningful sections of text. Coding was conducted by a single researcher (SB). Codes were derived inductively from the data and as analysis progressed these were organised into overarching or organising themes using NVivo 10 software. Data within themes were scrutinised for disconfirming and confirming views across the range of participants. Emerging themes were used to further develop the programme theories derived from the literature concerning how RMCs were intended to work and the contextual factors that influenced this. Analysis was led by SB in regular discussion with MR.

### Rigour

A number of steps were taken to ensure the trustworthiness of the study. Details derived from interviews on the organisation and functions of the schemes, were verified through a review of available relevant documentation and cross-checked with participants. Care was taken to ensure that the interviews conducted involved participants with a broad range of roles in the set-up, management and use of referral management centres and any identified differences between the views of interviewees were queried and explored. A draft of the analysis was shared with the majority of participants for feedback with respect to the accuracy of any quotes and factual details and interpretation of the findings. Emerging findings were also shared with members of the study advisory group for comment. To increase the likelihood of transferability of the findings, we took steps to ensure that the interview schedule enabled the collection of rich descriptive data on both the features of the schemes and the context in which they operated.

### Ethics, consent and permissions

Based on advice from the National Research Ethics Service and Cambridgeshire Local Research Ethics Committee, the study was deemed service evaluation and we did not obtain formal ethics approval, but sought to adhere to good research ethics practice throughout. Verbal consent was sought for participation in and audio-recording of interviews. Consent was sought for publication of anonymised quotes.

## Results

Two interviewers conducted 18 interviews across four sites. Between three and six respondents were interviewed at each site, drawing from the groups described above. Table 1 provides a summary of the characteristics of the schemes included and the candidates interviewed.

**Table 1:** Characteristics of participating referral management centres and interviewees

RMC	Key features	Interview number	Interviewee characteristics	Role in RMC
A	Functions: Clinical triage by GPs with additional expertise and allied health professionals; reviews referrals from 30 specialties; referrals either diverted to community service, returned to GP with advice or sent on to acute trust; manages patient bookings through 'choose and book' process; provides individual level advice and guidance to GPs, monthly referral reports and live access to referrals data; broader educational function Organisation: Provided by existing out-of-hours provider and software specialist; covers 3 CCGs, 95+ practices, 70 000 referrals; practices pay based on patient list size; operating 2010-present; patient booking originally provided out of area but now commissioned locally	A1	GP with managerial responsibility, director of RMS provider organization	Set-up and running
		A2	Manager, CCG	Commissioning
		A3	2 managers (joint interview), RMS provider	Set-up and running
		A4	GP	User only
		A5	GP	User only
B	Functions: Clinical triage by GPs; referrals either diverted to community service, returned to GP with advice or sent on to acute trust; reviews referrals from selected specialties; manages appointments booking (not linked to 'choose and book'); collects data to inform commissioning; limited individual level feedback; educational sessions and newsletters Organisation: Provided by existing out-of-hours provider; covers 1 CCG, 70+ practices; funded by CCG - cost per letter triaged; operating 2010-present; began as purely administrative with later introduction of GP triaging	B1	Manager, CCG	Running
		B2	GP with managerial responsibilities	Commissioning/set-up
		B3	GP with managerial responsibilities	Running and user
		B4	GP with managerial responsibilities	Development, running and user
		B5	GP with managerial responsibilities, also employed by RMS provider	Set-up and running, GP triager and user
		B6	GP	User only
C	Functions: No clinical triage by RMC staff; consultant triage commissioned from secondary care; following consultant triage referrals to selected specialties diverted to community service, returned to GP with advice or accepted by acute trust; non-clinical staff at RMC manage patient bookings through ‘choose and book’ and the process of sending referrals for selected specialties for consultant triage; collects data to inform commissioning; data reports at GP/practice level; internal peer review and educational sessions Organisation: Provided by existing educational services provider; covers 1 CCG, <20 practices; funded by CCG - unit cost per triaged and non-triaged referral; operating 2012-present; began as purely administrative with later introduction of consultant triage element	C1	Manager, RMS provider organization	Set-up and running
		C2	Manager, CCG	Commissioning and set-up
		C3	GP with managerial responsibility	Set-up, running and user
		C4	Practice Manager, also involved in management of RMS provider organisation	User, set-up and running
D	Functions: Clinical triage by GPs and nurses under supervision; focus on changing referral behavior through feedback and education; no diversion of referrals, GP retains responsibility for referral destination; no management of patient bookings; collects rich data; provides individual level feedback on referrals, weekly ‘top-tips’, access to detailed referral data; broader educational function Organisation: Provided by CIC set up specifically to deliver RMS; covers one CCG, opt in required – 16/24 participated; operating 2010–2013 – contract not continued.	D1	Manager, RMS provider	Set-up and running
		D2	GP with managerial responsibility, CCG	Commissioning
		D3	GP, also employed by RMS provider	GP triager and user

The four RMC interventions included in the study, all had among their aims the reduction of referrals to secondary care and shared a number of features: providing a central point of contact for GPs and service providers with regard to referrals; managing referrals to a wide range of specialties; collecting and analysing data and providing feedback and education to GPs. There were also considerable differences between the schemes, with respect to scale, the model used for clinical triage and the role played in managing appointment bookings and in diverting referrals.

The interviews conducted provided a rich source of data. Stakeholders described a range of aims and intended functions, (often concurrent within a single RMC, and evolving or being reprioritised over time), from which we could elicit a number of different programme theories. In addition, three themes were identified that related to the context in which the programmes were implemented: the impact of practical and administrative difficulties; the importance and challenge of stakeholder buy-in; and the dependence of perceived effectiveness on the aims and priorities of the scheme. The programme theories elicited and contextual factors impacting on implementation and delivery are explored in turn below.

### Programme theories

#### Diverse aims and functions reflect a range of underlying programme theories

**Fig. 1:**
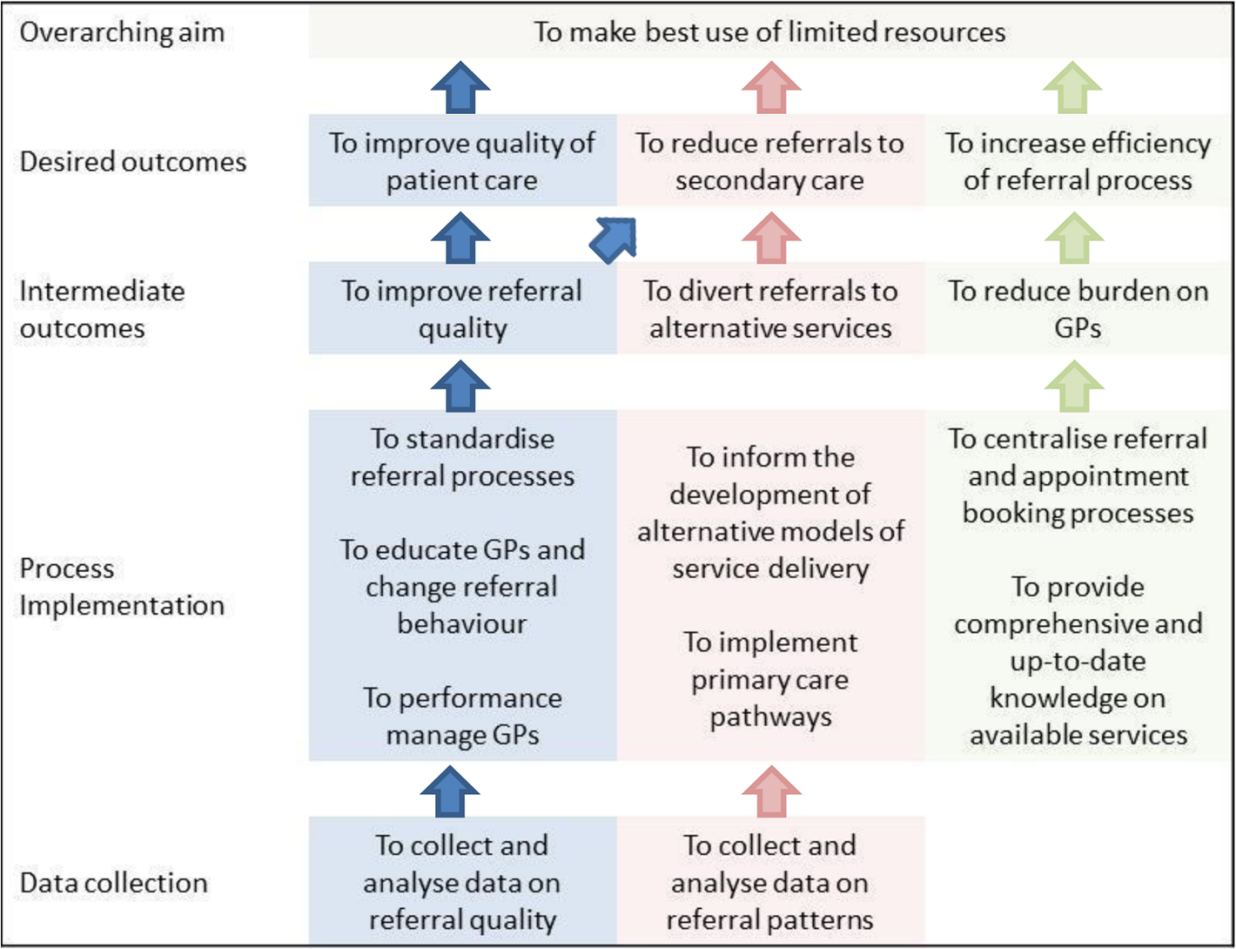
The aims and functions of referral management centres, based on interview findings

The RMCs included in this study were selected on the basis that they were intended to reduce referrals. In all cases, however, this was just one of many inter-related aims articulated (summarised in Fig. 1). At the highest level, interview data highlighted the overarching programme theory that **RMCs work by ensuring the best use of limited resources.** Three sub-theories were identified, relating to the ways in which this could be achieved: **i)RMCs improve the quality of referrals and patient care; ii) RMCs reduce referrals to secondary care; and iii) RMCs increase efficiency in the referral process.**

With respect to the mechanisms underlying quality improvement, interviewees described how RMCs aimed to enable the collection and analysis of data on referral quality, for these data to provide managers and GPs involved in managing and implementing the schemes with improved understanding of quality issues, and based on this, to enable standardisation of referral processes, education of GPs and implementation of primary care pathways. Desired outcomes included improvement in the quality (and reduction in the variability) of referrals and, in turn, improvements in the quality of patient care, with patients seen ‘*in the right place, at the right time, and by the right clinician or specialty*’ [B4, GP with managerial responsibilities], ensuring that ‘*when they’re sitting in front of that person, that person has everything that it’s reasonable and possible for them to have in order for them to treat that patient appropriately.*’ [B1, manager].

In order to change patterns of referral, interviewees described how RMCs aimed to enable the collection of rich data on referral patterns, to inform the development of alternative, more cost-effective models of service delivery to meet identified local needs, to divert referrals to alternative services (such as lower tariff community services) and, in so doing, to reduce referrals to secondary care. Also described, was the expectation that improving referral quality would also result in fewer referrals, with the aim of educating GPs to provide ‘*better quality letters, where more information was there, and therefore justifying more effectively, the reason for the referral*’ leading to GPs ‘*doing more in the general practice setting*’ [D3, GP Triager]

Finally, to make the referral process more parsimonious, RMCs aimed to allow the centralisation of referral and appointment booking processes, to hold comprehensive and up-to-date knowledge on available provider services, to reduce the (administrative and knowledge seeking) burden on referring GPs and through these measures to increase the efficiency of the referral process.

Interviewees described a complex network of aims and underlying tacit assumptions regarding how achievement of one aim depends on the delivery of another.

**D1, manager, RMC provider:***[the aim was] to improve the quality of referrals, to reduce unwanted variation and to trust that by doing those two things an unplanned consequence would be […] a reduction in unnecessary expenditure.*

**A2, manager, commissioning CCG:***Yeah, certainly my thrust has always been about improving the quality [of referrals] […] They can’t try and address the referral problem and not raise the quality of primary care*

The weight given to the different aims not only varied significantly between schemes, but also between stakeholders within the schemes. While a GP user of a scheme [B6] perceived the aim to be to ‘*save money on referrals into secondary care*’, a manager involved in its commissioning [B1] was clear that it aimed *‘to collect data on why patients are referred’* to inform how best to *‘shape services for the future’*. Similarly, a manager for an RMC provider organisation, described a mismatch between commissioner and provider expectations.

**C1, manager, RMC provider:***[the CCG] saw the RMC as a way of reducing referrals, therefore reducing costs. So I had to explain to them “you’re not going to reduce costs by just commissioning a RMC in isolation”, for starters.* […] *you will only reduce costs by commissioning community pathways at a reduced tariff. […] I have to be very clear when commissioners come to us about what their expectations are of a referral management centre […]*

#### The functions of RMCs and the programme theories underlying them evolved substantially over time

Not only did the aims of the schemes (or the weighting of them) vary between sites and between stakeholders, but interviewees also reported that they had evolved over time, dependent on changing local priorities, and on the successes already achieved.

**B3, GP with managerial responsibilities:***So the idea has changed as the service changed. They were initially collecting that data [on referral quality] and being able to present that [to practices] and want to possibly performance manage GPs.[…] but more recently it’s been about making sure that patients, are seen in the right place, so not going into inappropriate clinics. Not going to clinics with investigations that could have been done in primary care, having to be done again in secondary care. It’s about improving the patient experience, that’s the intention.*

Interviewees described how the functions of RMCs had evolved considerably since their initial implementation, in response to changes in aims and local context. In some instances, the original ambitions of the RMCs had led to changes in the local context within which they were operating, which, in turn had led to changes in the function of the RMC itself. For example, for two of the schemes (RMCs B and C), data collected by the RMC were reported to have informed the development of new community services, which in turn led to a need for the introduction of clinical triage as part of the RMC (to divert referrals appropriately).

**C1, manager, RMC provider:***…first of all […] we’d look at which specialties we thought would benefit from a community pathway, then we had to bring in the triage so it could be agreed which patients went into that community pathway. […] No changes have been made because the Gateway wanted change if you see what I mean, they were all changes that were going on in the landscape with the CCG and the GPs, the providers, and then the Gateway responds.*

Also highlighted was the fact that evolution in the functions and processes of RMCs resulted from efforts made to overcome identified challenges e.g. to respond to GP concerns, or iron out administrative glitches. For RMC A, for example, the bookings management element of the scheme was originally provided by an administrative team some distance away from the CCGs served, but for issues of cost, the acknowledged benefits and increased acceptability of using staff with local knowledge, this was relocated in area.

Capacity to evolve was described by several participants as key to the success of RMCs.

**B2, GP with managerial responsibilities:***[…] if it’s going to be a success, it needs to evolve,[…] you can start off with something but if it stays the same, then I think in the case of ours, we’d be having problems with it; whereas as its evolved it’s been able to respond to the needs of firstly the PCT and now the CCG.*

One interviewee acknowledged that, by encouraging appropriate referral behaviour on the part of GPs, RMCs might find that they were no longer needed.

**B1, manager, CCG:***[…] once the provider landscape has settled down, and people are much clearer about what they should be managing, and what they shouldn’t be managing, and what they should be sending, and what they shouldn’t be sending. If all that were in place, we might not need an RMS […] it might not be something that we’ll have forever.*

### Contextual factors

#### The impact of practical or administrative difficulties

Participants reflected on how practical issues had a profound impact on the functioning of the schemes, with difficulties often attributed to the need to manage evolving or unclear aims and functions.

##### Software and system limitations

System design and IT compatibility issues were reported to present a major challenge. Both a lack of clarity in aims and changes in the primary function of RMCs (e.g. from administrative to clinical function) presented IT challenges, with adaptations of software for new purposes causing difficulties for data capture and issues with the subsequent quality (and hence utility) of the data collected.

**B5, GP with managerial responsibilities, and GP triager:***I think that the problem was that the original set off of the RMS, being admin managed, had a database that was designed to manage from the admin point of view. […] So the clinical steps that were taken have been put on top of the old admin database. […] the impact that that made meant that data protection was a bit harder, but also that audit of clinical information was harder.*

**C2, manager CCG:***[…] it was an aspiration really, saying “we will use this data for commissioning intentions, commissioning purposes”. However, on looking at it, we’ve seen a few issues in the data quality, so we’re not prepared to use that as a reliance really. It’s good at giving a ball-park at the moment but we’ve tasked our data teams [at the CCG] to start, well, continue looking at it with them and get them up to speed as to what exactly we’re wanting to get out of it.*

Some features of clinical and administrative IT systems were highlighted as being important enablers of the schemes and conversely, their absence as disadvantageous. For RMC B, the lack of a facility for GPs to carry out triage remotely, was cited as a reason for difficulties experienced in recruiting local GP triagers, which in turn was reported to reduce the credibility of the scheme, affecting GP buy-in.

##### Contract and capacity issues

A number of interviewees described difficulties with successful contracting of RMC services and managing capacity within RMCs, in the context of continual and unpredictable changes in service structure and demand, with the potential to have a negative impact on both the sustainability of the scheme and the quality of patient care.

**C4, practice manager also involved with RMC:***[…] more triage specialties were brought in, […] That did result in a number of changes to process […] The team kept saying “you are adding extra steps in, you are increasing the risk of delays”, because it took longer to invest the time to do each referral. […] But that was the way the commissioners wanted it. So we were taking longer to process the referrals, the [RMC] team doubled in size […], which meant a lot of new members joined it and then it’s a five week training programme, so that hindered progress. And the activity levels started to rise too. So the combination of all those factors made it very difficult for the team to keep up with the volume that was coming in and a backlog started to generate.*

#### The importance and challenge of stakeholder buy-in and sustaining relationships

The most frequently cited challenge to the success and sustainability of RMCs was the need to achieve and sustain buy-in from the various stakeholders (commissioners, RMC providers, acute and community service providers and perhaps most importantly, referring GPs) on whose collaboration the proper functioning of the schemes depends.

##### Establishing shared vision

Both commissioners and providers of RMCs described a lack of clarity in the aims at the outset; with one interviewee commenting that those developing the RMC had been ‘*shooting in the dark*’ [C1, manager, RMC provider]. Also highlighted was the challenge of managing the differing priorities of commissioners and referring GPs, and communicating effectively to sell the concept to all involved.

**D1, manager, RMC provider:***We had to speak a little bit with fork tongue […] They [the CCG] were most interested in reducing the referrals but we said to them that, “If we speak to our constituency [GPs] about that subject we will get yawns and non-participation.”*

Unclear and poorly communicated aims were associated with a perceived lack of awareness among referring GPs, regarding the purpose of the intervention and of their role in it.

**B5, GP with managerial responsibilities, employed by RMC as triager:***If I was going to do this again, there would be a huge advertising campaign [targeting referring GPs], so to speak, actually saying what the intentions are […] I don’t really think that many of my colleagues really had a grasp of that at the beginning.*

**B6, referring GP:***As time’s gone on I think I’ve understood the aims, but it hasn’t been clearly set out by the RMS. I think I’ve learnt through the process what the aims are, but that’s my idea of what it is. I haven’t ever received anything from the RMS to say this is what we’re trying to do.*

A lack of early and effective involvement of GPs in the development of the aims of RMCs and a perception of being *‘told what to do’* [B6, GP] was reported to contribute to misinformation and distrust and consequently, resistance to engagement with RMC processes.

**B5, GP with managerial responsibilities, employed by RMC as triager:***[…] if I had a conversation with doctors about actually what it is that we’re trying to do, then I often find that people are much more ready to come on board, more aware of the process. And that one to one dialogue, it would be much better if that dialogue had been done at an earlier phase. When you have misinformation out there, then it pollutes what people actually think and turns it into a purely financial driven motive and actually the points about improving patient care, reducing waiting times and saving money at the same time will get missed.*

##### Sustaining engagement

Participants described a wide range of measures implemented to keep GPs on board (Table 2).

**Table 2:** Approaches to achieving and sustaining buy-in to referral management centres from referring GPs

Challenges	Approaches to achieving and sustaining buy-in from referring GPs
Lack of awareness among referring GPs of the aims and purpose of the scheme	• Engaging GPs in dialogue during the development of the scheme
	• Practice outreach through roadshows/practice visits
	• Opportunity to be involved as a triager
	• Regular newsletters/educational sessions on common referral issues
Cynicism and mistrust among GPs with respect to the achievements of the scheme	• Piloting systems and presenting evidence of success
	• Performance management of RMCs to ensure quality of patient care is not affected
Resistance to changing referral behaviour	• Offering incentives for referring through the RMC
	• Presenting bespoke data to practices at level of individual GPs to enable benchmarking
Frustration with bureaucracy	• Ensuring parsimony in administrative processes, e.g. evolving to include all specialties
	• Ensuring GPs are kept up to date with changes to processes through regular communication/newsletters etc.
Challenge to clinical autonomy	• Moving from purely administrative to clinical triage (based on the assumption that feedback from a fellow clinician would be better received than that from ‘*some manager or clerical person*’ [Int. B4])
	• Taking the approach of changing referral behaviour through education alone (with GPs retaining ultimate responsibility for referral destination)
	• Providing feedback to GPs on their referrals that supports education and learning
	• Ensuring that the tone of this feedback is moderate and advisory

The evolving nature of RMCs, however, was reported to present significant challenges, with rapid and widespread changes occurring simultaneously in many different referral pathways, leading to frustration among referring GPs, unable to keep up.

**B6, referring GP:***[…] the rules changed. I think, initially, it was some clinics, then it was other clinics and then it was everything and it was keeping up with their change […] As they were developing, we were trying to understand what they were doing.*

Clarity and parsimony of referral processes were described to be important factors influencing GP support.

**A4, referring GP:***[…] the advantage is that now we don’t do Choose and Book it is a lot more straightforward at that interface with the patient; […], so we saved a lot of time.*

**C3, GP with managerial responsibility:***[…]as a GP I don’t want to be thinking about a lot of different pathways. Ideally I want to try and keep it down to as little as possible, so you know the more the RMC does, the better, because you just, you end up sending 90–95 % of your referrals to the RMC and you don’t have to think about it after that.*

Maintaining quality and safety in patient care was considered paramount to ensuring continued engagement. The potential for introducing delay into the referral process was acknowledged as a serious threat to the acceptability of the schemes.

**B2, GP with managerial responsibilities:***We knew that the whole systems would start falling apart if clinical safety and quality fell apart […] When GPs heard about it [breaches in agreed turnaround times for referrals] … I mean it wasn’t kept secret… then it was that loss of faith in something […].*

##### Respecting GP autonomy and responsibility

A major stumbling block in selling the concept of RMCs to GPs, was the perception that such schemes present a challenge to clinical autonomy, avoidance of which was cited by interviewees across all the included schemes as an important consideration in the design, implementation and sustainability of the initiatives (as outlined in Table 2). Even with respect to the RMC that took a purely educational approach however, with GPs retaining responsibility for referral destination, interviewees described challenges associated with GP resistance to negative feedback, highlighting the need to consider not only what feedback is given but also how it is delivered.

**D1, manager, RMC provider:***the tone in which the triagers rattled their notes into the computer, when they were finding a referral not as good as it might be, was not always as diplomatic as it could be. […] we had to put that text through a moderator who would change all the language so that there were no […] unnecessary spikes in it, […] We train them [GP triagers] all in how to do that.*

**D2, GP with managerial responsibilities:***there is rivalry in primary care. We all like to feel our practices are the best practice […]. I know that a number of practices were being contacted fairly frequently […] they hadn’t done this, that or the other; and I think that some practices didn’t like that and left, which were the very practices one could say that really required that support.*

#### The dependence of perceived effectiveness on the aims and priorities of the scheme

All interviewees reported that the RMC they described was successful with respect to at least one of its aims. The perceived effectiveness of the schemes, however, varied by stakeholder and according to how the aims and priorities were specified.

**B2, GP with managerial responsibilities:***[…] if you say that the initial thing that a referral management service set-out to achieve [was] the information, and where were the key areas that had the highest number of referrals, actually it achieved that […] [I]t was then set up to say “well, if you have that data is there actually anything that you can do?” Now it didn’t say you’ve got to do this, ‘cause actually that would have tied its hands and you would have had either failure or success. So because it wasn’t tied down too much, you were then able to move into the next phase[…]*

##### Different measures of success for different stages of RMC development

Perceived success achieved in relation to the aims set out in Fig. 1 could be seen to reflect, to some degree, the maturity of the scheme, as, for example, effective data collection and analysis was a prerequisite for the implementation of processes to achieve the specified intermediate and desired outcomes and move towards the overarching aim to ensure best use of NHS resources. Interviewees tended to report greater difficulties in measuring achievements against (and were less confident in their claims with respect to) higher level goals, with success claimed less consistently, reflecting marked disagreements between stakeholders (as illustrated in Table 3).

**Table 3:** Perceived success of RMCs in relation to specified aims

	Outcome	RMC A	RMC B	RMC C	RMC D
Overarching aim:	Better use of resources	✓ (A1, A2, A3)	? (B1, B3) - Too soon to draw conclusions	? (C2) - Too soon to draw conclusions	✓ (D1, D3) ✘ (D1, D2, D3)- Commissioner not convinced by figures
Desired outcomes:	Improved quality of patient care	✓ (A1, A3, A5) - Patients generally unaware ✘ (A2, A5) - Occasional restricted choice for patients - Concern about quality of community service provision	✓ (B2, B5) - Community clinics offer convenience and shorter waiting times ✘ (B2, B3, B4, B6) - Reduced patient choice - Referrals sometimes get lost	✓ (C1, C2) - Quicker referral times - More time and information available to support patient choice	✓ (D1, D3) - Patients unaware - RMC highlights cases that should be upgraded to urgent
	Reduced referrals to secondary care	✓ (A1, A2, A4) - Up to 15 % reduction	✓ (B4) - 8 % reduction	? (C2) - Not a significant impact but too soon to tell	✓ (D1, D3) ✘ (D2) - Commissioner not convinced by figures
	Improved efficiency of referral process	✓ (A2, A3, A4, A5) ✘ (A1, A2, A5) - Some duplication of work due to administrative glitches - Teething trouble with IT/systems	✓ (B3, B6) ✘ (B1, B2, B3, B4) - Some issues with under-capacity and turnaround times - Some duplication of work	✓ (C1, C2, C3, C4) ✘ (C1, C3, C4) - Some issues with under-capacity and turnaround times - Issues with booking management system	N/a
Intermediate outcomes:	Improved referral quality	✓ (A2, A3)	? (B1, B4) - Believed to be reducing variability in referrals	? (C1) - Anecdotally, fewer rejections from providers	✓ (D1, D3)
	Diversion of referrals to alternative services	✓ (A2, A4)	✓ (B3, B5)	✓ (C3)	N/a
	Reduced burden on GPs and practice staff	✓ (A4, A5)	✓ (B3)	✓ (C1, C2, C3, C4)	N/a
Process implementation:	Standardised referral processes	✓ (A2, A3)	Not mentioned in data	✓ (C1, C2)	N/a
	GP education/culture change	✓ (A1, A3)	✓ (B2, B4, B5, B6)	✓ (C1, C2, C3, C4)	✓ (D1, D2, D3)
	Implementing primary care pathways	✓ (A2)	✓ (B1)	✓ (C2)	✘ (D2) - Did not effect pathway change as expected
	Centralising referral/booking processes	Not mentioned in data	✓ (B4, B6)	✓ (C1, C2)	N/a
	Providing up-to-date service knowledge	✓ (A1, A2, A4)	✓ (B1, B3)	✓ (C1, C2)	✓ (D3)
	Informing service development	Not mentioned in data	✓ (B2, B4, B5, B6)	✓ (C1) ✘ (C2) - Some concerns regarding data quality	Not mentioned in data
Data:	Collection and analysis of data	✓ (A2)	✓ (B2, B3, B4)	✓ (C3, C4)	✓ (D2, D3)

✓ One or more participants describe success in achieving stated aim; ✘ One or more participants describe a lack of success in or concerns regarding achievement of stated aim; ? One or more participants describe being unsure or not yet ready to reach a conclusion on achievement of stated aim. Participant identifier codes are provided in parenthesis. Explanatory supporting examples are also provided. Since RMC D did not aim to involve direct management of referral process, participants did not describe achievement with respect to related aims (thus coded as n/a – not applicable)

For all schemes, RMC providers and commissioners expressed with varying degrees of confidence that referrals to secondary care were being curbed by the schemes. Calculating efficiency savings, however, was reported to be a highly complex process, requiring sophisticated analysis, factoring in costs of running the RMC, the provision of alternative services to which referrals were diverted, and possible disinvestments in secondary care. Providers and commissioners described the challenges of both calculating savings and communicating the findings, which were thus open to interpretation.

**B1, manager CCG:***[…] we’ve got a schedule [for calculating savings] […], in the absence of anything more specific, we’re trying to quantify it that way. Now whether there’s a better or more sophisticated way of doing it, I don’t know […] I think we’ll always be looking at our data, and how it’s presented, and whether there’s a more accurate way of looking at things. But, I think on the whole it’s convincing people that it’s a good thing, and it’s the right thing.*

##### The trouble with differing priorities

Effectively communicating success and a shared vision of what this looks like for a particular scheme was held by many to be essential for its survival. The provider of one scheme recounted how its failure to meet the commissioner’s aim to centralise and standardise referral processes (due to a number of practices opting out), combined with a lack of confidence in the figures demonstrating cost-effectiveness, meant that despite strong evidence of success (from the perspective of the providers), the contract for the scheme was not renewed.

**D1, manager, RMC provider:***[the CCG] took the decision […] that it was more important to bring all the practices together under a common denominator, even if it was the lowest common denominator, rather than to […] look at the evidence and purchase that which is known to be effective. We were bitterly, bitterly disappointed… […] we were very well resourced, we fulfilled all our expectations, we got going, we saw the curve beginning to bend […] The big, big problem was that we as leaders didn’t operate in such a way that others felt drawn in […]*

## Discussion

### Summary of findings

Professionals involved in the commissioning and provision of RMCs, and GP users of the schemes, described the wide range and evolving nature of their aims and functions. Practical and administrative difficulties, compounded by the need for schemes to evolve to meet changing needs, were reported to have a significant impact on their successful functioning. Achieving buy-in from and sustaining relationships between RMC stakeholders was both challenging (partly as a result of a lack of clarity in aims and implementation issues) and key to success. The perceived effectiveness of schemes, however, was dependent on their aims and priorities. Many schemes were judged successful by those involved, with reference to a range of outcomes (e.g. the collection of useful data, GP education and centralised and streamlined referral processes) despite limited evidence of reduced referral rates or cost savings.

### Strengths and limitations

This is the first qualitative study to focus specifically on functioning RMCs. Building on work exploring approaches to referral management more broadly [4, 14] this study includes schemes experiencing a range of degrees of success and sustainability (three had identified problems of one kind or another and implemented their own solutions and the fourth, although deemed successful with respect to reducing referrals, had closed down), and a wide range of stakeholders, sampled across and within RMCs of varying scope and operational structure. This allowed access to a broad range of perspectives, to explore the ways in which RMCs are intended to work and to seek to understand challenges to implementation and factors influencing perceived success. Steps taken to ensure the rigorous conduct of the study (such as data triangulation), mean that we are confident in the trustworthiness of the findings. While coding of interview data was conducted by a single researcher, which increased risk of subjective bias, the emergent coding framework was regularly discussed between the researchers conducting the interviews and the wider research team, and member checks performed with study participants to validate the findings. There are, however, a number of further limitations to the study. Since only a small number of participants were interviewed in relation to each of the schemes some of the findings may represent idiosyncratic views. In particular, it is important to note that many of the stakeholders interviewed had considerable investment in the success of their scheme. This may have led participants to overestimate or overplay the benefits of the schemes, meaning that findings with respect to the perceived effectiveness should be interpreted cautiously with this in mind. Furthermore, the fact that our sites were essentially self-selected means that they may not represent the experiences of a wider range of referral management centres, a fact which may limit the transferability of the results.

### Discussion of findings

The wide range of aims and diverse models of operation of RMCs highlighted in this study is in keeping with observations previously reported in the literature [4, 7, 14]. The evolving nature of RMCs, has also been noted [4, 14]. Our study suggests that this occurs as stakeholders respond to the challenges that emerge throughout the process of implementing RMCs, by modifying their aims and the ways in which they function. In other words, they actively adapt RMCs to meet the demands of local circumstances. Our study also indicates that there is a feedback loop between the context in which the programme occurs and the programme itself; for example, through being educated by the RMC on what is or is not appropriate to refer, GPs learn what constitutes an ‘appropriate’ referral or learn about additional facilities available in the community, and begin to send referrals that are more in line with what providers perceive they should be receiving. This in turn means that fewer referrals are diverted or rejected by the RMC, which can lead to a revision of the aims and function of the RMC [17]. The evolution of aims was also reported to influence the type of information that the RMC required, so, as functions evolved, IT systems sometimes made it more difficult to collect the data that RMCs needed to fulfil their renewed functions. Our finding that the aims of RMCs evolved over time is consistent with the tenet of realist evaluation that complex interventions are not universally successful but have a pattern of different outcomes depending on context. Measuring the success of RMCs is also further complicated by our findings that different stakeholders were not clear what the criteria for success were and specified multiple criteria for success, some of which were more valued by one set of interests than another. This means that one group of stakeholders can describe the RMC as a success by selectively focusing on one set of criteria and ignoring others.

The importance and challenge of stakeholder engagement in ensuring the success of RMCs have been highlighted in previous studies, with a clear focus on quality [4] and the provision of good data [14] identified as important ways to overcome cynicism and mistrust and generate support. A key difference between the findings of this and other studies, however, is that concerns expressed about the centres with respect to issues such as introducing delays in treatment, interfering with the GP’s clinical judgement and restricting patient choice [3, 4, 14] emerge as challenges to be overcome rather than inherent flaws in the functioning of the schemes. Our findings are consistent with those of Pawson et al. [20], which show that clinical buy-in is gained if clinicians are given the power to have significant control over the aims and functioning of the RMC, which in turn means that the RMC’s remit meets clinical interests rather more than managerial ones. Our findings are also consistent with work that shows the tension between the managerial and clinical roles being adopted increasingly by clinicians following the most recent NHS reforms [21]. Sometimes, two sets of interests appeared to jar against each other, leading to a lack of clarity and confusion about the aims of the RMCs and potentially hindering their functioning. While GPs were in general sceptical and sometimes resistant to feedback, how feedback was given and by whom appeared to have a significant impact on how GPs responded to RMCs. Feedback could foster GP engagement when provided in a constructive or educational way, but lead to disengagement if provided in a critical or punitive manner, in line findings relating to the use of peer review in general practice more broadly [22].

With respect to the success or effectiveness of RMCs, this study was not designed to identify the impact of RMCs on patterns of referral. Nevertheless, reflecting the limited and equivocal published evidence from elsewhere on the effectiveness of RMCs [4, 7, 14] in most sites we found that interviewees were hesitant to draw conclusions with respect to the ability of the schemes to deliver on the aim to make better use of resources. This hesitancy appeared to result, in the main part, from the difficulties described in calculating, interpreting and communicating efficiency savings, requiring complex analysis, taking into account a broad range of outcomes and associated costs and savings (costs of running the RMC, the provision of alternative services to which referrals were diverted, and possible disinvestments in secondary care). For all the RMCs at least one stakeholder described improved patient care as an outcome, and a reduction in referrals to secondary care was reported for three out of four schemes. However, the leap required to explain how this represented better use of resources overall was seldom made, despite this being reported as an explicit aim by one or more stakeholders for all of the RMCs. In addition, the common sentiment that it was too soon to draw conclusions, could be seen to reflect the fact that it takes time for the effects of the RMC to filter through to the wider use of resources within the health system and that a number of schemes had been up and running for only a relatively short period of time.

As well as considering the impact of RMCs on referral patterns, previous research has also suggested that such schemes improve referral quality [23], in keeping with this, our data suggest that the perception that RMCs improved referral quality was widely held by a range of stakeholders. This is an area where further research is clearly needed. In addition to understanding the overall impact of RMCs on patterns of referral, it is important to determine their impact on GPs’ decisions whether to refer or not, which type of clinician the person needs to be referred to, and which local services can provide that care.

## Conclusions

The findings of this study have a number of implications for the development of similar schemes both in the UK and internationally. First, clarity of aims and shared understanding between stakeholders are essential to get engagement and buy-in, and this necessitates the early involvement of GPs in the development of the schemes. Second, while indicators of success should be agreed between stakeholders from the outset, it needs to be acknowledged that schemes are likely to change over time. Third, the evolution of schemes needs to be anticipated and plans made for potential modifications to referral processes including IT systems, and for effective communication of changes to relevant stakeholders.
